# Photocontrolled apoptosis induction using precursor miR-664a and an RNA carrier-conjugated with photosensitizer

**DOI:** 10.1038/s41598-021-94249-7

**Published:** 2021-07-22

**Authors:** Kazunori Watanabe, Tomoko Nawachi, Ruriko Okutani, Takashi Ohtsuki

**Affiliations:** 1 Graduate School of Interdisciplinary Science and Engineering in Health Systems, Okayama University, 3-1-1 Tsushimanaka, Okayama, 700-8530 Japan; 2 Department of Biomedical Engineering, Faculty of Engineering, Okayama University, 3-1-1 Tsushimanaka, Okayama, 700-8530 Japan

**Keywords:** Biomaterials, Gene delivery, Nucleic-acid therapeutics, RNA, Cell death

## Abstract

Methods to spatially induce apoptosis are useful for cancer therapy. To control the induction of apoptosis, methods using light, such as photochemical internalization (PCI), have been developed. We hypothesized that photoinduced delivery of microRNAs (miRNAs) that regulate apoptosis could spatially induce apoptosis. In this study, we identified pre-miR-664a as a novel apoptosis-inducing miRNA via mitochondrial apoptotic pathway. Further, we demonstrated the utility of photoinduced cytosolic dispersion of RNA (PCDR), which is an intracellular RNA delivery method based on PCI. Indeed, apoptosis is spatially regulated by pre-miR-664a and PCDR. In addition, we found that apoptosis induced by pre-miR-664a delivered by PCDR was more rapid than that by lipofection. These results suggest that pre-miR-664a is a nucleic acid drug candidate for cancer therapy and PCDR and pre-miR-664a-based strategies have potential therapeutic uses for diseases affecting various cell types.

## Introduction

Apoptosis is a programmed cell death that elicits no inflammatory responses. Thus, methods to spatially induce apoptosis can be useful for treatments such as cancer therapy^1^. To control the induction of apoptosis, methods that use light, such as photodynamic therapy (PDT) and photochemical internalization (PCI), have been developed. For instance, PDT, which uses a combination of a photosensitizer and harmless light to generate reactive oxygen species, induces photo-dependent apoptosis^2,3^. Methods combining PDT and small interfering RNA (siRNA) have also been developed to obtain synergistic effects^4,5^.

PCI, which uses a photosensitizer and harmless light, delivers targeted molecules such as drugs, peptides, and nucleic acids into the cytosol^6–8^. We developed a PCI-based apoptosis-inducing molecule, TatBim-Alexa. This molecule, which is a conjugate of the Tat cell-penetrating peptide, the BH3 domain from the apoptosis-inducing protein Bim, and the photosensitizer Alexa, can spatially control apoptosis, depending on photo-activation^9,10^.

MicroRNAs (miRNAs) have been attracting attention as nucleic acid drugs^11^. miRNAs are small non-coding RNAs that play important roles in the posttranscriptional regulation of gene expression by targeting the 3′-untranslated regions (UTRs) of mRNA^12^. Primary-miRNA (pri-miRNA), which consists of a stem-loop structure, is transcribed by RNA polymerase II, and cleaved by Drosha-DiGeorge Syndrome Critical Region 8 (Drosha-DGCR8) complex to produce precursor miRNA (pre-miRNA)^12–14^. Then, pre-miRNA is exported from the nucleus and cleaved by Dicer to produce 21–23 base pair mature miRNA^12,14^. Importantly, miRNAs regulate biological and physiological processes such as apoptosis, proliferation, and cell differentiation^11,15,16^. For instance, miR-15 and miR-16 induce apoptosis by suppressing the anti-apoptotic B cell lymphoma 2 protein^17^. In addition, miR-466 induces apoptosis by downregulating the Runt-related transcription factor, which is associated with osteoblast differentiation and inhibits tumor growth in vivo^18^.

We developed a photoinduced cytosolic dispersion of RNA (PCDR) method, which is an intracellular RNA delivery method^8,19,20^. The PCDR method is based on the PCI mechanism and can photo-dependently deliver RNA into the cytosol via the endocytic pathway. In other words, the PCDR method can spatially regulate RNA delivery into the cytosol. In this study, we used the PCDR method to deliver apoptosis-inducing miRNA and thereby spatially regulate apoptosis. In addition, we compared the apoptosis induction achieved via the PCDR method and that induced by lipofection.

## Methods

### Preparation of TatU1A-Alexa546 molecules

TatU1A protein was prepared as described previously^19^. Purified TatU1A protein and Alexa Fluor 546 C5 (Life Technologies, CA, USA), which contains a thiol-reactive maleimide group, were mixed and then incubated at 25 °C for 1 h. The Alexa Fluor 546-modified TatU1A molecules (TatU1A-Alexa546) were purified in a Centri-Sep spin column (Princeton Separations, NJ, USA) equilibrated with T buffer containing 20 mM Hepes–KOH (pH 7.6), 115 mM NaCl, 5.4 mM KCl, 1.8 mM CaCl_2_, 0.8 mM MgCl_2_, and 13.8 mM glucose. Protein concentrations were determined using a Protein Assay Kit (Nacalai Tesque, Kyoto, Japan). The TatU1A labeling efficiency was calculated by measuring Alexa Fluor 546 absorbance. Then, labeling efficiency was adjusted to 35% using unlabeled TatU1A protein.

### Preparation of pre-miR-664a, pre-miR-664a-*U1A*, and pre-miRNA-*U1A* control

In this study, pre-miR-664a, pre-miR-664a-*U1A*, and pre-miRNA-*U1A* control (non-targeting pre-miRNA containing the *U1A* recognition sequence) were prepared by in vitro transcription. To generate DNA templates for transcription, primer extension was performed using 2 µM of each primer (shown in Table S1) in a reaction mixture containing KOD Dash DNA polymerase (TOYOBO, Osaka, Japan). The resulting template DNA was precipitated with 2-propanol. T7 RNA polymerase was prepared as described previously^21^. The transcription reaction was carried out at 37 °C for 4 h in a reaction mixture containing 40 mM Tris–HCl (pH 8.0), 24 mM MgCl_2_, 5 mM dithiothreitol, 10 mM guanosine monophosphate, 2 mM of each NTP, 1.8 U/mL inorganic pyrophosphatase (Sigma, St. Louis, MO, USA), 26.2 µg/mL purified T7 RNA polymerase, and 10 µg/mL DNA template. The pre-miRNA transcripts were purified using an 8% denaturing polyacrylamide gel. All pre-miRNAs were renatured by incubating for 1 min at 85 °C, followed by slow cooling to 4 °C.

The FAM-labeled RNA (RNA-FAM) was purchased from Hokkaido System Science (Hokkaido, Japan). The RNA-FAM sequence was as follows: 5′-GAU UAU GUC CGG UUA UGU ACA UUG CAC UCC GUA CAU AAC CGG ACA UAA UCdT dT-FAM-3′ (the U1A binding sequence is underlined).

### Cell culture

HeLa cells were obtained from RIKEN BRC, which participates in the National Bio-Resource Project of the MEXT, Japan. HeLa cells were maintained in RPMI 1640 medium (Nacalai Tesque) with 10% heat-inactivated fetal bovine serum (FBS) (Sigma) and 1% antibiotic–antimycotic solution (Gibco, Gaithersburg, MD, USA) at 37 °C and 5% CO_2_.

SH-SY5Y cells (DS Pharma Biomedical, Osaka, Japan) were seeded onto collagen-coated dishes (Corning, Cambridge, MA, USA) and maintained in Dulbecco’s modified Eagle’s medium-F12 (1:1) medium (Nacalai Tesque) supplemented with 10% heat-inactivated FBS and 1% penicillin–streptomycin (Gibco) at 37 °C and 5% CO_2_.

Flp-In-293 cells (Thermo Fisher Scientific, Waltham, MA, USA) were seeded onto collagen-coated dishes and maintained in Dulbecco’s modified Eagle’s medium (Nacalai Tesque) supplemented with 10% heat-inactivated FBS and 1% penicillin–streptomycin at 37 °C and 5% CO_2_.

### Cellular RNA delivery by lipofection

The miRNA-664a-5p mimic and miRNA mimic control were purchased from Dharmacon (Lafayette, CO, USA). The cells were transfected with 10 nM miRNA-664a-5p mimic or 10 nM pre-miR-664a using Lipofectamine RNAiMAX (Invitrogen, Carlsbad, CA, USA) for 24 h at 37 °C and 5% CO_2_. For control experiments, the cells were also transfected with miRNA mimic control or pre-miRNA-*U1A* control. The next day, the medium was replaced with fresh growth medium or differentiation medium containing 30 µM retinoic acid (Wako Pure Chemical Industries, Osaka, Japan). The cells were again transfected with miRNA mimics, pre-miR-664a, or pre-miRNA-*U1A* control in growth medium or differentiation medium after 2 days. After 24 h, the medium was replaced with fresh growth medium or differentiation medium, and the cells were cultured for an additional 2 days.

To induce apoptosis, the cells were transfected with 10 nM pre-miR-664a or pre-miR-664-*U1A* using Lipofectamine 3000 (Invitrogen) for 24 h at 37 °C and 5% CO_2_. For control experiments, the cells were also transfected with pre-miRNA-*U1A* control.

### Cellular RNA delivery by PCDR

TatU1A-Alexa546 (2 µM) and pre-miRNA or RNA-FAM (0.2 µM) were mixed in Opti-MEM (Gibco). The cells were grown in 96-well plates and treated for 4 h with TatU1A-Alexa546/RNA complexes prepared as described above. After washing with growth medium, HeLa cells were irradiated with 530–550 nm light at 20 J/cm^2^ intensity using a mercury arc lamp passed through a MWIG mirror unit and a 40×, 20×, or 4× objective lens using a fluorescence microscope (IX51, Olympus, Tokyo, Japan). SH-SY5Y cells were irradiated with 530–550 nm light at 30 J/cm^2^. After photo-irradiation, the cells were grown for 24 h at 37 °C and 5% CO_2_.

Confirming whether the RNA-FAM was delivery efficiently depended on the photo-irradiation energy, the cells were irradiated with 530–550 nm light from 0 to 40 J/cm^2^.

### Detection of apoptotic cells by Nucview488 caspase-3 Assay Kit

Apoptosis was detected using NucView488 caspase-3 Assay Kit (Biotium, Freemont, CA, USA). Apoptotic cells were visualized using an Olympus IX51 fluorescence microscope, and 0.25 µM staurosporine (Wako Pure Chemical Industries) was used as a positive control.

### Immunocytochemistry

HeLa cells were transfected with 10 nM pre-miR-664a or pre-miR-664-*U1A* using Lipofectamine 3000 for 24 h at 37 °C and 5% CO_2_. Immunocytochemistry was performed as described previously^16^. Mouse anti-Cytochrome *c* (dilution 1:300) was purchased from Cell Signaling Technology (Danvers, MA, USA). Alexa 594-conjugated secondary antibody was purchased from Invitrogen, and 4′,6-diamidino-2-phenylindole (DAPI) was obtained from Dojindo. The stained cells were examined using an Olympus IX51 fluorescence microscope.

### Mitochondrial permeability transition pore opening

HeLa cells were transfected with 10 nM pre-miR-664a or pre-miR-664-*U1A* using Lipofectamine 3000 for 20 h at 37 °C and 5% CO_2_. Transfected HeLa cells were treated with HBSS containing 1 µM calcein-AM (Dojindo, Kumamoto, Japan) and 5 mM cobalt chloride, which quenched the cytosolic and nuclear fluorescence of calcein, for 20 min at 37 °C and 5% CO_2_. Cells were washed with HBSS, and then were observed using an Olympus IX51 fluorescence microscope.

### Detection of TatU1A-Alexa546 in endosomes using Lysotracker green

TatU1A-Alexa546 (2 µM) and pre-miRNA-*U1A* control (0.2 µM) were mixed in Opti-MEM. The cells were grown in 96-well plates and treated for 4 h with TatU1A-Alexa546/RNA complexes prepared as described above. Opti-MEM containing those complexes was replaced with fresh Opti-MEM containing Lysotracker green (Thermo Fisher Scientific) for 1 h at 37 °C. Cells were washed twice with Opti-MEM, and then observed using an Olympus IX51 fluorescence microscope.

### Detection of polarization of mitochondrial membranes

HeLa and SH-SY5Y cells were transfected with 10 nM pre-miRNA-*U1A* control and pre-miR-664-*U1A* using Lipofectamine 3000 for 24 h at 37 °C and 5% CO_2_. To observe the polarization of the mitochondrial membrane, the JC-1 mitochondrial membrane potential assay kit (Cayman Chemical, Michigan, USA) was used according to the manufacturer’s instructions. The fluorescence images of JC-1 aggregates [red] and monomers [green] were examined using a fluorescence microscope (IX-51). The fluorescence intensities were calculated using CellSens software (Olympus).

### RNA-FAM incorporation

The cells were transfected with RNA-FAM using Lipofectamine 3000 or PCDR, as described above. RNA-FAM mean fluorescence intensity within transfected cells was determined by flow cytometry using a Guava easyCyte flow cytometer (Merck Millipore, Burlington, MA, USA). For all experiments, data were acquired from 15,000 cells.

To observe endosomal escape in SH-SY5Y and HeLa cells, RNA-FAM taken up by cells was visualized using an Olympus IX51 fluorescence microscope.

### Cell viability in cells transfected with miRNA or pre-miRNA

Cell viability was evaluated using the Cell Counting Kit-8 (Dojindo). After transfection, the medium was replaced with fresh medium containing CCK-8 solution. The absorbance at 450 nm was measured to determine cell viability.

### Time-lapse imaging of lipofection- and PCDR-induced apoptosis

Time-lapse imaging was used to observe apoptotic cells. To perform the experiment under the same conditions as the PCDR method, HeLa cells were pre-treated for 4 h with Opti-MEM and transfected with 10 nM pre-miRNA using Lipofectamine 3000. Apoptotic cells were observed at 12, 14, 16, 18, 20, 22, and 24 h after the transfection reagent was added.

TatU1A-Alexa546 (2 µM) and pre-miRNAs (0.2 µM) were mixed in Opti-MEM. HeLa cells were treated with TatU1A-Alexa546/RNA complexes for 4 h. After washing, the cells were irradiated with 530–550-nm light at 20 J/cm^2^ using a mercury arc lamp passed through the MWIG mirror unit and 40 × objective lens of a fluorescence microscope. Apoptotic cells were observed at 4, 6, 8, 10, 12, 14, 16, 18, and 24 h after photo-irradiation.

### Statistical analysis

The number of apoptotic cells was counted using CellSens software. All results are expressed as means ± SEM. R^22^ and EZR^23^ software were used for the statistical analyses. The number of the experimental replication is shown in the respective figure legends.

## Results and discussion

### Pre-miR-664a induces apoptosis

**Figure 1:**
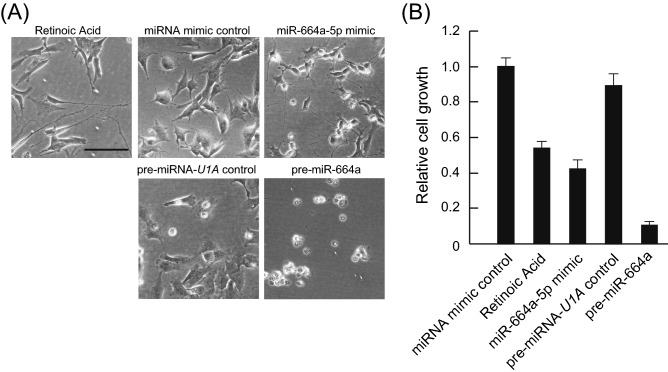
Pre-miR-664a inhibits growth and induces cell death in SH-SY5Y cells. (**A**) Phase contrast images of SH-SY5Y cells transfected with miR-664a-5p, pre-miR-664a, miRNA mimic, or pre-miRNA-*U1A* control using Lipofectamine RNAiMAX. Retinoic acid induced SH-SY5Y cell differentiation. Scale bars indicate 100 µm. (**B**) The viability of SH-SY5Y cells transfected with miR-664a-5p, pre-miR-664a, miRNA mimic, or pre-miRNA-U1A control using Lipofectamine RNAiMAX. Cell viability was normalized to the viability of cells treated with miRNA mimic control. Data represent the means ± SEM of three independent experiments.

We previously showed that miR-664a-3p promotes neuronal differentiation of SH-SY5Y cells^16^. When we analyzed the neuronal differentiation mechanism induced by miR-664a-5p, interesting results were obtained. Retinoic acid and miR-664a-5p mimic induced neuronal differentiation of SH-SY5Y cells (Fig. 1A). In contrast, cell death was observed in cells transfected with pre-miR-664a, which is the precursor of miR-664a-5p. In addition, pre-miR-664a inhibited cell growth of the cells (Fig. 1B).

**Figure 2:**
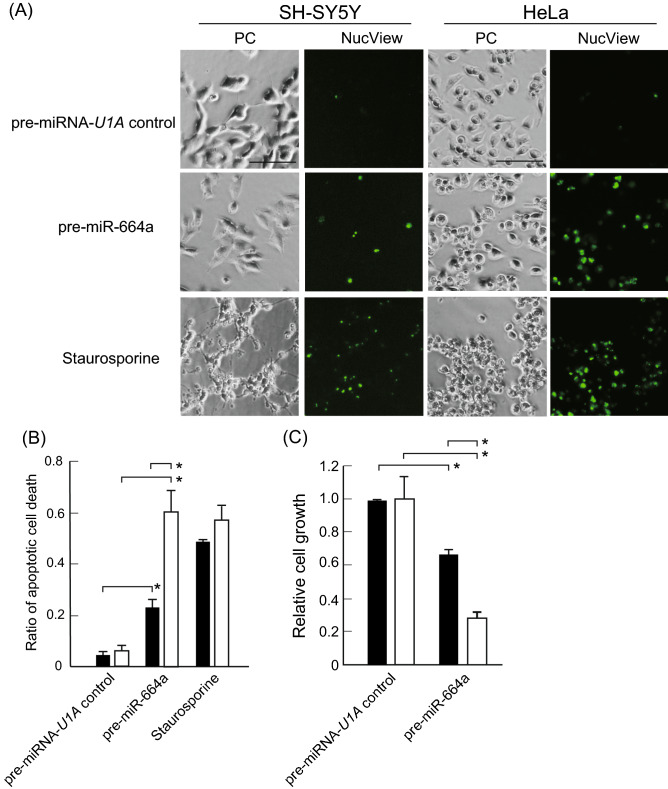
pre-miR-664a delivered by lipofection induces apoptosis. (**A**) SH-SY5Y and HeLa cells were transfected with pre-miRNA-*U1A* control or pre-miR-664a using Lipofectamine 3000. Apoptotic cells were detected using NucView 488 caspase assay kit. Staurosporine was used as a positive control. *PC* phase contrast images. Scale bars indicate 100 µm. (**B**) Apoptosis efficiency of SH-SY5Y (black bar) and HeLa cells (white bar) are shown. Data represent the means ± SEM of five independent experiments. *P < 0.01; two-way ANOVA and Tukey’s multiple comparisons test. (**C**) The growth of SH-SY5Y (black bar) and HeLa cells (white bar) transfected with pre-miRNA-*U1A* control or pre-miR-664a using Lipofectamine 3000. Data represent the means ± SEM of five independent experiments. *P < 0.05; P-values were calculated using two-way ANOVA and Tukey’s multiple comparisons test.

To confirm whether pre-miR-664a induced apoptosis, apoptotic cells were evaluated using a NucView 488 caspase-3 assay kit, which can detect apoptosis. As shown in Fig. 2A,B, the ratio of apoptotic cells was significantly increased in SH-SY5Y cells transfected with pre-miR-664a using Lipofectamine 3000 compared to those transfected with pre-miRNA-*U1A* control (0.23 vs. 0.04, respectively). The ratio of apoptotic cells was also significantly increased in HeLa cells transfected with pre-miR-664a compared to the pre-miRNA-*U1A* control (0.53 vs. 0.06, respectively). In addition, SH-SY5Y and HeLa cell growth was inhibited by pre-miR-664a treatment, compared to pre-miRNA-*U1A* control (Fig. 2C).

Nucview488 detects only the final caspase3/7 activity. Therefore, apoptosis of the cells treated with pre-miR-664a was detected using JC-1 dye reagent, which detects depolarization of the mitochondrial membrane. As shown in Fig. S1, the JC-1 fluorescence ratio in SH-SY5Y cells treated with pre-miR-664a was lower compared to that in the cells treated with pre-miRNA-*U1A* control. In addition, the JC-1 fluorescence ratio in HeLa cells treated with pre-miR-664a was also lower compared to that in the cells treated with pre-miRNA-*U1A* control (Fig. S1), suggesting that pre-miR-664a induced apoptosis.

Furthermore, we observed cytochrome *c* (cyt-*c*) localization and measured mitochondrial permeability transition pore (MPTP) opening, to detect apoptosis induced by pre-miR-664a. Cyt-*c* localizes into the intermembrane space of mitochondrial membrane under the standard condition, and cyt-*c* releases from the mitochondria to the cytosol due to MTPT opening during apoptosis^24,25^. To observe cyt-*c* localization, we performed immunostaining of cyt-*c*. Cyt-*c* in the cells treated with pre-miRNA-*U1A* control was observed as dots in the cytoplasm (Fig. S2). In contrast, cyt-*c* in the cells treated with pre-miR-664a was diffused in the cytoplasm, and the nuclear fragmentation and condensation were observed. To determine whether pre-miR-664a induced MPTP opening, MPTP opening was measured by calcein/Co^2+^-quenching method. The fluorescence intensity of calcein-AM in the cells treated with pre-miR-664a was lower than that in the cells treated with pre-miRNA-*U1A* control, suggesting that pre-miR-664a induces MPTP opening (Fig. S3). These results suggest that pre-miR-664a induced MPTP opening to promote the release of mitochondrial cyt-*c*. In summary, these results suggest that pre-miR-664a is a novel miRNA that induces apoptosis via mitochondrial apoptotic pathway.

Pre-miR-664a generates miR-664a-5p and miR-664a-3p. Previous reports showed that miR-664a-5p promotes neuronal differentiation of SH-SY5Y cells and promotes osteogenic differentiation of human bone marrow-derived mesenchymal stem cells^16,26^. miR-664a-3p promotes cell proliferation in gastric cancer cells^27^. In addition, apoptosis induction by miR-664a-3p or miR-664a-5p alone has not been reported. Therefore, both miR-664a-3p and miR-664a-5p are likely to be important for the induction of apoptosis.

Regarding potential pre-miR-664a targets relating to apoptotic pathway, siRNA-mediated knockdown of high mobility group AT-hook 2 (HMGA2), which is a target of miR-664a-5p, induces apoptosis in PC3 and DU145 cells^26,28^. Therefore, apoptosis may be induced by miR-664a-5p-mediated down-regulation of HMGA2. In addition, caspase 3/7 activity of HMGA2- and checkpoint kinase 1 (CHK1)-knockdowned cells is higher than that of HMGA2-knockdowned cells^29^. CHK1 regulates cell cycle progression and cell cycle arrest induced by DNA damage^29^. The targetscan7.2 software^30^ predicts that CHK1 is not a target of miR-664a-3p, however multiple proteins regulated cell cycle progression and cell cycle checkpoint such as cyclin A are target. Hence, pre-miR-664a may induce apoptosis via down-regulations of HMGA2 by miR-664a-5p and cell cycle progression regulated by miR-664a-3p.

### Pre-miR-664a-*U1A* induces apoptosis

**Figure 3:**
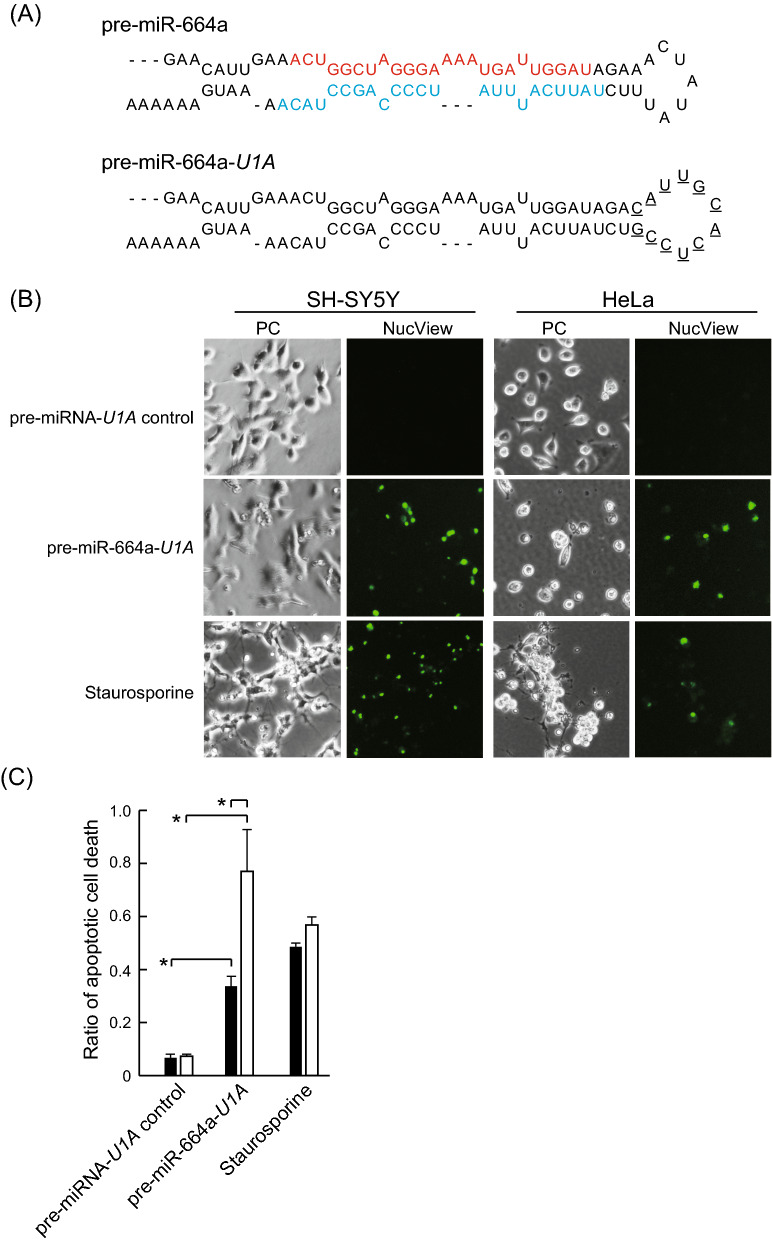
Pre-miR-664a-*U1A* delivered by lipofection induces apoptosis. (**A**) The pre-miR-664a and pre-miR-664a-*U1A* sequences are shown. The underlined sequence shown in pre-miR-664a-*U1A* is replaced with the U1A binding sequence. The red font sequence indicates miR-664a-5p. The light blue font sequence indicates miR-664a-3p. (**B**) SH-SY5Y and HeLa cells were transfected with pre-miRNA-*U1A* control or pre-miR-664a-*U1A* using Lipofectamine 3000. Apoptotic cells were detected using NucView 488 caspase assay kit. Staurosporine was used as a positive control. *PC* phase contrast images. Scale bars indicate 100 µm. (**C**) Apoptosis efficiency of SH-SY5Y (black bar) and HeLa cells (white bar). The data represent the means ± SEM of five independent experiments. *P < 0.01; P-values were calculated using two-way ANOVA and Tukey’s multiple comparisons test.

We attempted to control region-specific apoptosis induction by combining pre-miR-664a and the PCDR method. However, the PCDR method using the RNA carrier U1A protein can only deliver RNA containing a U1A recognition sequence^19^. Therefore, we prepared pre-miR-664-*U1A*, where the pre-miR-664a hairpin structure was replaced with the U1A recognition sequence (Fig. 3A). To confirm whether pre-miR-664-*U1A* induced apoptosis, we elevated apoptosis of HeLa cells and SH-SY5Y cells transfected with pre-miR-664-*U1A* using Lipofectamine 3000. As shown in Fig. 3B,C, pre-miR-664-*U1A* induced apoptosis in HeLa cells (0.77) and SH-SY5Y cells (0.33). In addition, apoptosis efficiency by pre-miR-664a was similar to that by pre-miR-664a-*U1A* in HeLa and SH-SY5Y cells (Fig. S4). These results indicate that substituting U1A recognition sequence in pre-miR-664a has hardly any effect on apoptosis induction.

### Pre-miR-664a-*U1A* delivered by PCDR method induces apoptosis

**Figure 4:**
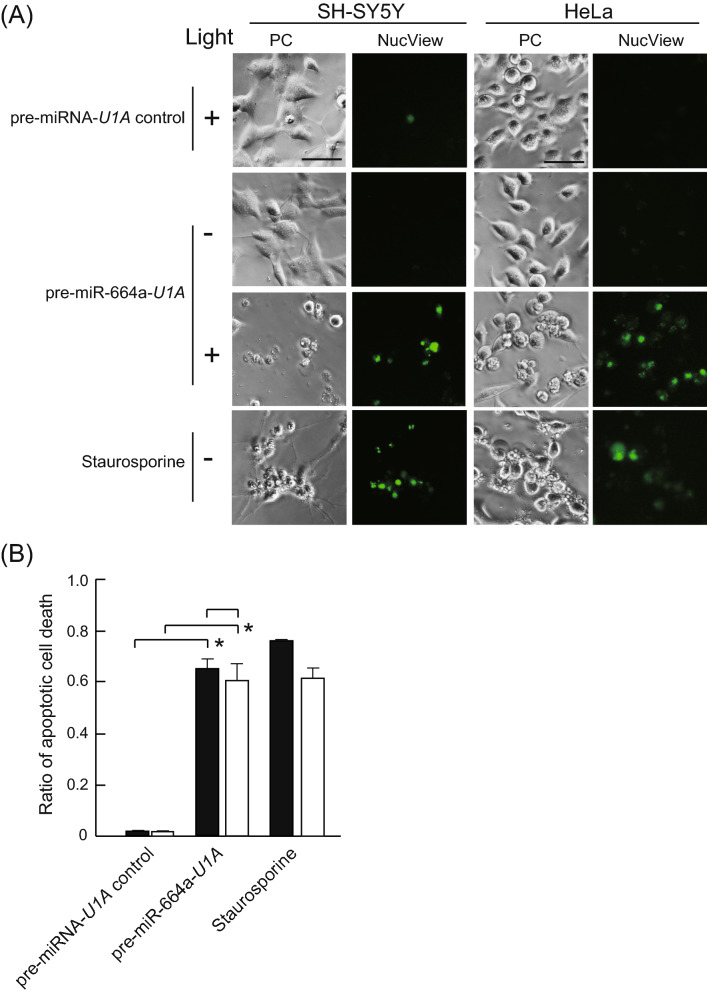
pre-miR-664a-*U1A* delivered by photoinduced cytosolic dispersion of RNA (PCDR) induces apoptosis. (**A**) pre-miRNA-*U1A* control or pre-miR-664a-*U1A* were delivered into SH-SY5Y and HeLa cells using PCDR. Then, the cells were photo-irradiated. Apoptotic cells were detected using NucView 488 caspase assay kit. Staurosporine was used as a positive control. PC: Phase contrast images. Scale bars indicate 100 µm. (**B**) Apoptosis efficiency of SH-SY5Y (black bar) and HeLa cells (white bar). Data represent the means ± SEM of five independent experiments. *P < 0.01; P-values were calculated using two-way ANOVA and Tukey’s multiple comparisons test.

We next attempted to induce photo-dependent apoptosis by combining pre-miR-664a-*U1A* and the PCDR method in HeLa cells and SH-SY5Y cells. The cells transfected with pre-miR-664a-*U1A* using PCDR showed photo-dependent apoptosis (Fig. 4A,B). In contrast, without photo-irradiation, pre-miR-664a-*U1A* did not induce apoptosis in these cells. In addition, the cells transfected with pre-miRNA-*U1A* control by PCDR did not show apoptosis. These results indicate that pre-miR-664a-*U1A* delivered by the PCDR method induces photo-dependent apoptosis.

### Cell type-dependency of transfection efficiency of PCDR and lipofection methods

**Figure 5:**
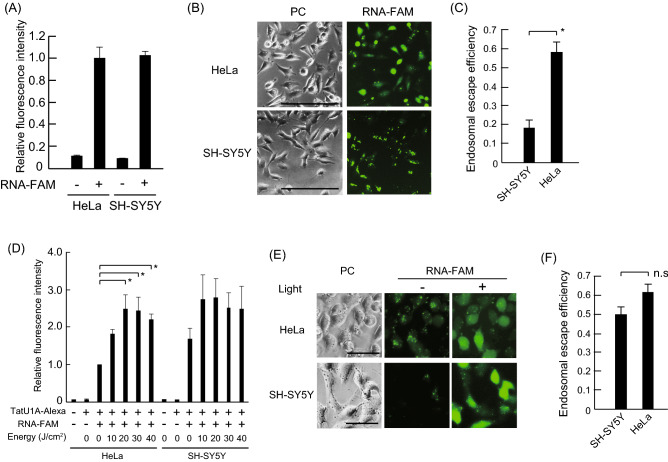
RNA-FAM incorporation in the cytosol using Lipofectamine 3000 and photoinduced cytosolic dispersion of RNA (PCDR). (**A**) The fluorescence intensity of RNA-FAM delivered into SH-SH5Y and HeLa cells using Lipofectamine 3000 was measured using flow cytometry. Data represent the means ± SEM of three independent experiments. The fluorescence intensity of HeLa cells transfected with RNA-FAM was defined as 1.0. (**B**) RNA-FAM was transfected into SH-SY5Y and HeLa cells using Lipofectamine 3000. RNA-FAM images were obtained after transfection. *PC* phase contrast images. Scale bars indicate 100 µm. (**C**) The endosomal escape efficiencies of RNA-FAM were calculated by counting the number of the cells in which FAM fluorescence was dispersed within the cytosol (N_F_) and the number of the cells (N_T_). The endosomal escape efficiency was defined as N_F_/N_T_. Data represent the means ± SEM of four independent experiments. *P < 0.05; P-values were calculated using one-way ANOVA and Dunnett’s test. (D) The fluorescence intensity of RNA-FAM delivered by PCDR into SH-SH5Y and HeLa cells was measured using flow cytometry. The fluorescence intensity of HeLa cells irradiated at 0 J/cm^2^ was defined as 1.0. Data represent the means ± SEM of five independent experiments. *P < 0.05; P-values were calculated using one-way ANOVA and Tukey’s test. (**E**) RNA-FAM was delivered by PCDR into the cytosol of SH-SY5Y and HeLa cells. RNA-FAM images were obtained before and after photoirradiation. SH-SY5Y and HeLa cells were irradiated at 30 J/cm^2^ and 20 J/cm^2^, respectively. *PC* Phase contrast images. Scale bars indicate 50 µm. (**F**) The endosomal escape efficiencies of TatU1A-Alexa546/RNA complexes were calculated by counting the number of the cells in which FAM fluorescence was dispersed within the cytosol after photoirradiation (N_F_) and the number of the cells (N_T_). The endosomal escape efficiency was defined as N_F_/N_T_. Data represent the means ± SEM of four independent experiments. *P < 0.05, not significant (n.s); P-values were calculated using one-way ANOVA and Dunnett’s test.

After determining that pre-miR-664a-*U1A* delivered by PCDR induces apoptosis, we evaluated whether the PCDR delivery method was more efficient than transfection using lipofectamine. Using the lipofection method, apoptosis in HeLa cells was higher than that in SH-SY5Y cells (Fig. 2B). However, apoptosis in HeLa cells was almost the same as in SH-SY5Y cells using the PCDR method (Fig. 4B). We speculate that apoptosis efficiency may depend on the RNA concentration delivered into the cells, which is a consequence of differences in the RNA delivery methods. In other words, the RNA concentration delivered into HeLa cells using the lipofection method may be higher than the RNA concentration delivered into SH-SY5Y cells, while the RNA concentration delivered via PCDR is similar in HeLa and SH-SY5Y cells. Therefore, we used flow cytometry to measure the incorporation of FAM-labeled RNA (RNA-FAM) into HeLa and SH-SY5Y cells by lipofection. The RNA-FAM fluorescence intensity in HeLa cells after lipofection was similar to the fluorescence intensity in SH-SY5Y cells (Fig. 5A). This suggests that the concentration of RNA-FAM delivered into HeLa and SH-SY5Y cells was similar, which cannot explain the difference in pre-miR-664a-*U1A*/lipofectamine-induced apoptosis between HeLa and SH-SY5Y cells.

Transfection efficiency by cationic lipids depends on the cell type^31^. Cationic lipids, including lipofectamine, form polyplexes and deliver RNA into the cells via the endocytic pathway. Therefore, the lipofectamine/RNA-FAM complex might be trapped in endosomes in a cell type-dependent manner. As shown in Fig. 5B,C, cytosolic RNA-FAM dispersion in HeLa cells was more efficient than in SH-SY5Y cells. These results suggest that the difference in cell type-dependent apoptosis efficiency after lipofection depends on the efficiency of RNA delivery into the cytosol rather than the whole cell, which includes endosomes.

The TatU1A/RNA complex enters cells via the endocytic pathway^19,32^. We confirmed that TatU1A-Alexa546/pre-miRNA-*U1A* control complexes accumulated into endosomes in SH-SY5Y and HeLa cells before photo-irradiation using Lysotracker green. Lysotracker green detects acidic membranous structures such as endosomes and lysosomes. These complexes were colocalized with Lysotracker green in SH-SY5Y and HeLa cells (Fig. S5), suggesting that TatU1A-Alexa546/pre-miRNA-*U1A* control complexes accumulated into endosomes via the endocytic pathway.

Singlet oxygen is generated from Alexa Fluor 546 during photo-irradiation, which allows the complex to escape from endosomes and disperse throughout the cytosol^32^. Therefore, the RNA concentration delivered into the cytosol via the PCDR method may be dependent on the applied light energy. We first examined whether the RNA concentration delivered into the cytoplasm was dependent on light energy. As shown in Fig. 5D, RNA-FAM fluorescence in HeLa cells was increased by photo-irradiation, and peaked at 20 J/cm^2^. RNA-FAM fluorescence in SH-SY5Y cells was slightly increased by photo-irradiation (Fig. 5D). In addition, the maximum RNA-FAM fluorescence intensity in HeLa cells was similar to that in SH-SY5Y cells, suggesting that the RNA concentration delivered into HeLa cells was approximately the same as that in SH-SY5Y cells. RNA-FAM fluorescence intensity increased after the TatU1A/RNA complex escaped from the endosome^32^. The increased RNA-FAM fluorescence intensity after photo-irradiation suggests that RNA-FAM was dispersed into the cytosol by photo-irradiation (Fig. 5D).

To observe the escape of RNA-FAM from endosomes, RNA-FAM was delivered into the cell by the PCDR method and visualized using a fluorescence microscope (Fig. 5E). RNA-FAM endosomal escape was observed in HeLa and SH-SY5Y cells (Fig. 5E,F). These results indicate that cell type-independent apoptosis by the PCDR method occurs because endosomal escape occurs in both HeLa and SH-SY5Y cells.

The TatU1A-Alexa546/pre-miRNA complex delivered by the PCDR method enters cells via the endocytic pathway. Singlet oxygen photogenerated from Alexa Fluor 546 facilitates the escape of the complex from endosomes^32^. Therefore, the endosomal escape efficiency induced by the PCDR method might be cell type-independent, unlike the lipofection method.

In summary, apoptosis induced by lipofection is cell type-dependent because RNA delivery into the cytosol depended on the cell type. In contrast, apoptosis induced by PCDR is cell type-independent because RNA delivery into the cytosol did not depend on the cell type.

### Apoptosis is more rapidly induced by PCDR than by the lipofection method

**Figure 6:**
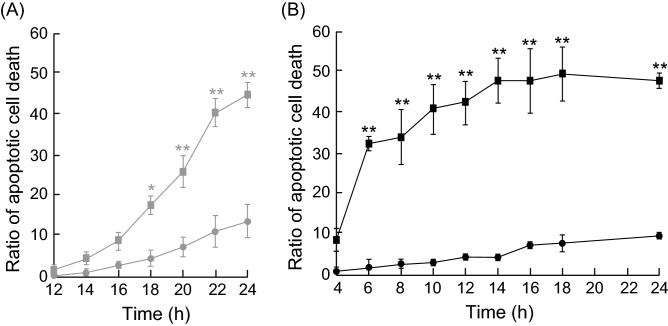
Ratio of time-dependent apoptosis in cells transfected by photoinduced cytosolic dispersion of RNA (PCDR) or lipofection. HeLa cells were transfected with pre-miRNA-*U1A* control or pre-miR-664a-*U1A* using Lipofectamine 3000 (**A**) or PCDR (**B**). Apoptotic cells were detected at 12, 14, 16, 18, 20, 22, and 24 h (lipofection) or 4, 6, 8, 10, 12, 14, 16, 18, and 24 h (PCDR). The ratio of apoptotic cells treated with pre-miRNA-*U1A* control by lipofection (gray circles), pre-miR-664a-*U1A* by lipofection method (gray squares), pre-miRNA control by PCDR (black circles), and pre-miR-664a-*U1A* by PCDR (black squares). Data represent the means ± SE of four (PCDR) or five (lipofection) independent experiments. *P < 0.05, **P < 0.01; P-values were calculated using two-way ANOVA and Tukey’s multiple comparisons test comparing pre-miR-664a-*U1A* with pre-miRNA-*U1A* control at each time point.

Our previous study showed that the concentration of cytoplasmic RNA delivered by PCDR rapidly increased compared to RNA delivery by lipofection (Lipofectamine 3000)^20^. Therefore, to examine whether apoptosis induced by PCDR occurred faster than that by lipofection, we observed apoptotic cells using time-lapse imaging. Apoptosis of cells transfected with pre-miR-664a-*U1A* via lipofection increased in a time-dependent manner, and was significantly induced at 18 h compared to pre-miRNA-*U1A* control (Fig. 6A). In contrast, apoptosis caused by pre-miR-664a-*U1A* and PCDR was significantly induced at 6 h compared to pre-miRNA-*U1A* control (Fig. 6B). Further, apoptosis was observed at 12 h in cells transfected with pre-miR-664a-*U1A* by PCDR, but not lipofectamine (Fig. 6A,B). Thus, apoptosis induced by PCDR occurred more rapidly than lipofection-mediated apoptosis.

### Region-specific apoptosis induction by the PCDR method

**Figure 7:**
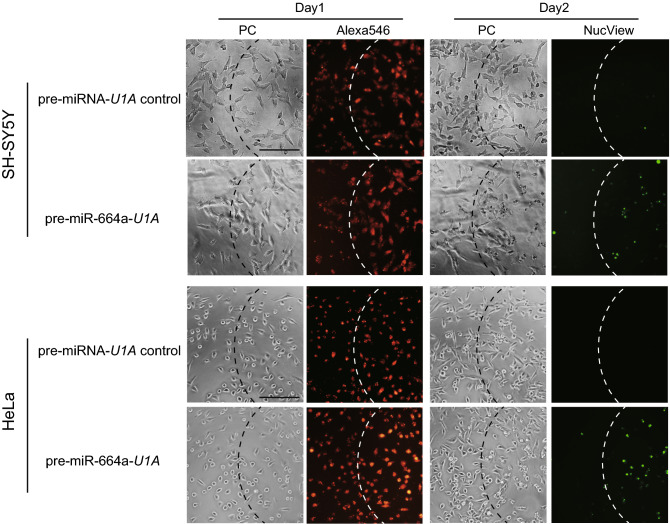
Region specific apoptosis of cells transfected with pre-miR-664a-*U1A.* The cells were treated with TatU1A-Alexa546 and pre-miRNA complexes. Then, the cells to the right of the dotted line were irradiated (Day 1). Phase contrast and TatU1A-Alexa546 fluorescence images were immediately obtained. Apoptotic cells were detected using NucView 488 caspase assay kits (Day 2). *PC* phase-contrast images. Scale bars indicate 100 µm.

We attempted to demonstrate region-specific apoptosis induction by combining pre-miR-664a-*U1A* and PCDR. After SH-SY5Y cells were treated with TatU1A-Alexa546/pre-miRNA complexes, the cells in a specific region were irradiated (Fig. 7, Day 1). TatU1A-Alexa546 entered the cytoplasm only within the photo-irradiated region. Twenty-four hours later, apoptosis was detected using NucView 488 (Fig. 7, Day 2). Apoptotic cells were observed only within the photo-irradiated region. Similar experiments were performed using HeLa cells. Apoptosis was induced only in photo-irradiated cells (Fig. 7). These results indicate that apoptosis can be spatially controlled by pre-miR-664a-*U1A* delivered by PCDR, regardless of cell type.

### Pre-miR-664a induces apoptosis in benign cells, Flp-In-293 cells

SH-SY5Y and HeLa cells, which are malignant cells, were used in the present study. We confirmed that pre-miR-664a induced apoptosis in Flp-In-293 cells, which were created from benign HEK293 cells. As shown in Fig. S6, pre-miR-664a induced apoptosis in Flp-In-293 cells. This result suggests that pre-miR-664a probably induces apoptosis in human cells. Therefore, a method for region-specific delivery of pre-miR-664a, as occurs in the PCDR method, is important for the application of pre-miR-664a in cancer therapy.

### Potential therapeutic uses of strategies based on PCDR and pre-miR-664a

The PCI method can effectively deliver targeted molecules to the targeted cells. For instance, pre-clinical studies suggest that PCI can effectively deliver chemotherapeutic agents into tumor cells and improve treatment efficacy^6^. Thus, a PCI strategy will likely develop as an effective cancer therapy.

The strategy of pre-miR-664a and PCDR based on PCI has potential therapeutic uses for cancer therapy. However, for this strategy to be successful, its problems must be addressed. Naked pre-miR-664a and TatU1A are unstable in blood, and TatU1A-Alexa/pre-miR-664a complexes may be distributed around the whole body because TatU1A has no target specificity. In order to use this method in vivo, subcutaneous or local injections are required. In addition, it will be important to improve the stabilities of pre-miR-664a and TatU1A in blood and the tumor specificity of TatU1A-Alexa546/pre-miRNA complex. For instance, the development of pre-miR-664a with nucleotide modifications^33^ and TatU1A with modifications^34^ are important for improving their stability in blood. In order to accumulate TatU1A-Alexa/pre-miR-664a complexes in a tumor, it is necessary to fuse TatU1A with a peptide which accumulates into specific cells, such as RGD peptide^35^.

We previously developed a nanocarrier named “lactosome”. Lactosome accumulated in mouse tumors through enhanced permeability and retention effects^36^. In addition, lactosome could photo-dependently deliver siRNA into the cells in vitro^37^. Therefore, the combination of pre-miR-664a and lactosome may induce apoptosis in a light-dependent manner in vivo.

## Conclusions

In this study, we identified pre-miR-664a as a novel apoptosis-inducing miRNA. Furthermore, apoptosis could be spatially regulated by pre-miR-664a-*U1A* delivered by PCDR. Therefore, pre-miR-664a is a potential nucleic acid drug candidate for cancer therapy. To understand apoptosis induction mechanisms by pre-miR-664a, further analyses of the target genes of pre-miR-664a will be needed.

We demonstrated that while apoptosis efficiency after lipofection depends on the cell type, apoptosis efficiency after PCDR is cell type-independent. Furthermore, apoptosis induced by PCDR occurs more rapidly than that induced by lipofection. The increased apoptosis rate using the PCDR method may be attributed to the rapid dispersion of RNA into the cytosol after photo-stimulation. Therefore, PCDR and pre-miR-664a-based strategies have potential therapeutic uses for diseases affecting various cell types.

## Supplementary Information

Supplementary Information.
