# Supplementary material for: Photocontrolled apoptosis induction using precursor miR-664a and an RNA carrier-conjugated with photosensitizer
Source: Sci Rep. 2021 Jul 22;11:14936. doi: 10.1038/s41598-021-94249-7 (PMC8298592; doi:10.1038/s41598-021-94249-7)
Supplement: Supplementary file 1 — Supplementary Information. [file 41598_2021_94249_MOESM1_ESM.pdf]

Supporting Information to:

Photocontrolled apoptosis induction using precursor miR-664a and an RNA carrier-conjugated with photosensitizer

Kazunori Watanabe[1, 2*], Tomoko Nawachi[1], Ruriko Okutani[2], Takashi Ohtsuki[1, 2]

[1] Graduate School of Interdisciplinary Science and Engineering in Health Systems, Okayama University, 3-1-1 Tsushimanaka, Okayama, 700-8530, Japan

[2] Department of Biomedical Engineering, Faculty of Engineering, Okayama University, 3-1-1 Tsushimanaka, Okayama, 700-8530, Japan

*Correspondence to: Kazunori Watanabe

Fax: +81-251-8219; Tel: +81-251-8220.

Email address: k_watanabe@okayama-u.ac.jp

Table S1 List of primers used in the present work for transcription of pre-miRNA

Name	Sequence
pre-miR-664-Fw	5'-CGA AAT TAA TAC GAC TCA CTA TAG AAC ATT GAA ACT GGC TAG GGA AAA TGA TTG GAT AGA AAC TAT ATT TCT ATT CAT TTA TCC CC-3'
pre-miR-664-Re	5'- TTT TTT CAT TTT GTA GGC TGG GGA TAA ATG AAT AGA AAT ATA GTT TCT ATC-3'
pre-miR-664-*U1A*-Fw	5'-CGA AAT TAA TAC GAC TCA CTA TAG AAC ATT GAA ACT GGC TAG GGA AAA TGA TTG GAT AGA CAT TGC ACT CCG TCT ATT CAT TTA TCC CC-3'
pre-miR-664-*U1A*-Re	5'-TTT TTT CAT TTT GTA GGC TGG GGA TAA ATG AAT AGA CGG AGT GCA ATG TCT ATC C-3'
pre-miRNA-*U1A* control-Fw	5'-CGA AAT TAA TAC GAC TCA CTA TAG AGC GAC TAA ACA CAT CAA CAT TGC-3'
pre-miRNA-*U1A* control-Re	5'-AAG AGC GAC TAA ACA CAT CAA CGG AGT GCA ATG TTG ATG TGT TTA GTC-3'

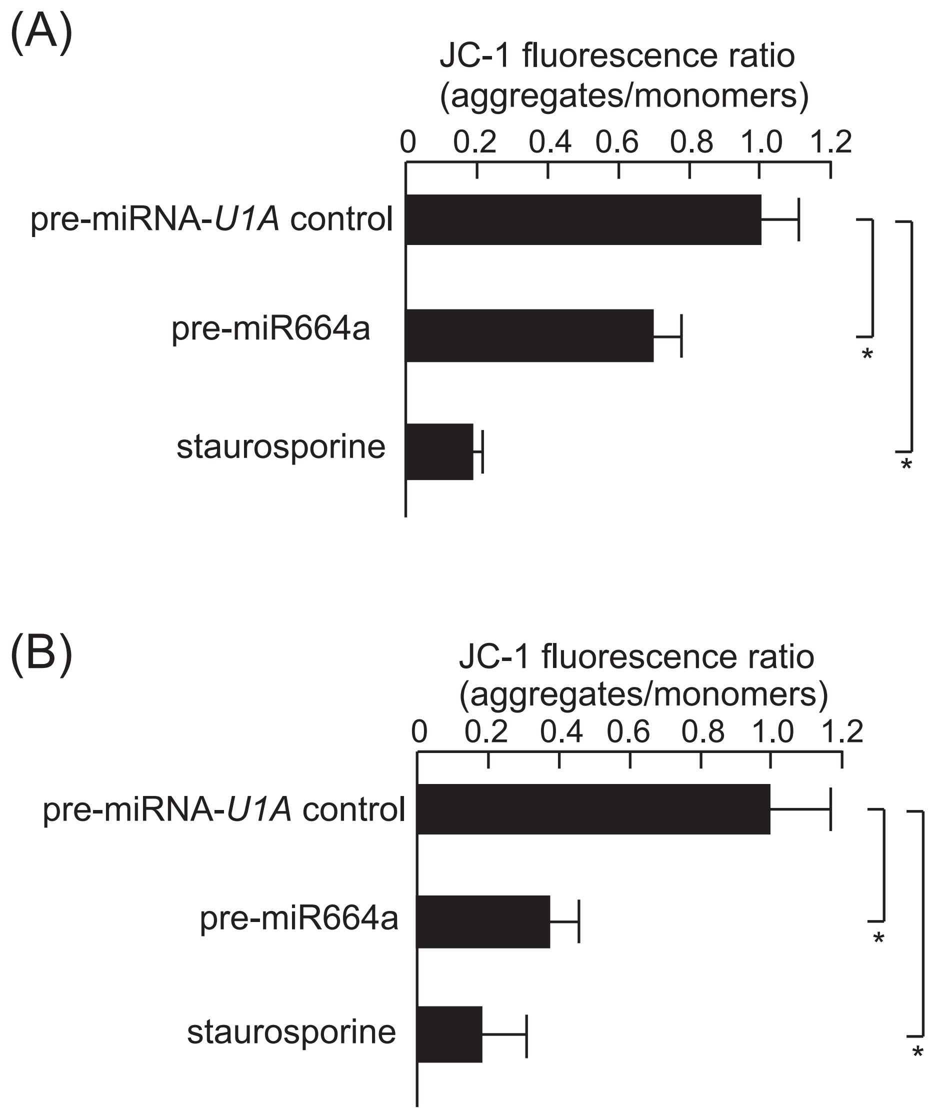

Figure S1. Detection of apoptosis by using JC-1 dye in the cells treated with pre-miR-664a.

(A) SH-SY5Y cells were transfected with pre-miRNA-*U1A* control or pre-miR-664a at 37˚C for 24 h in an atmosphere of 5% CO_2. Data represent the means ± SEM of four independet experiments. (B) HeLa cells were transfected with pre-miRNA-*U1A* control or pre-miR-664a at 37˚C for 24 h. Apoptosis was detected usign JC-1 dye. The fluorescence images of JC-1 aggregates [red] and monomers [green] were examined using fluorescence microscope. JC-1 fluorescence ratio of the cells transfected with pre-miRNA-*U1A* control was used as a control for 1.0. 0.25 μM staurosporine was used as a positive control. Data represent the means ± SEM of three independent experiments. *P<0.05; P-values were calculated using one-way ANOVA and Tukey' s multiple comparisons test.

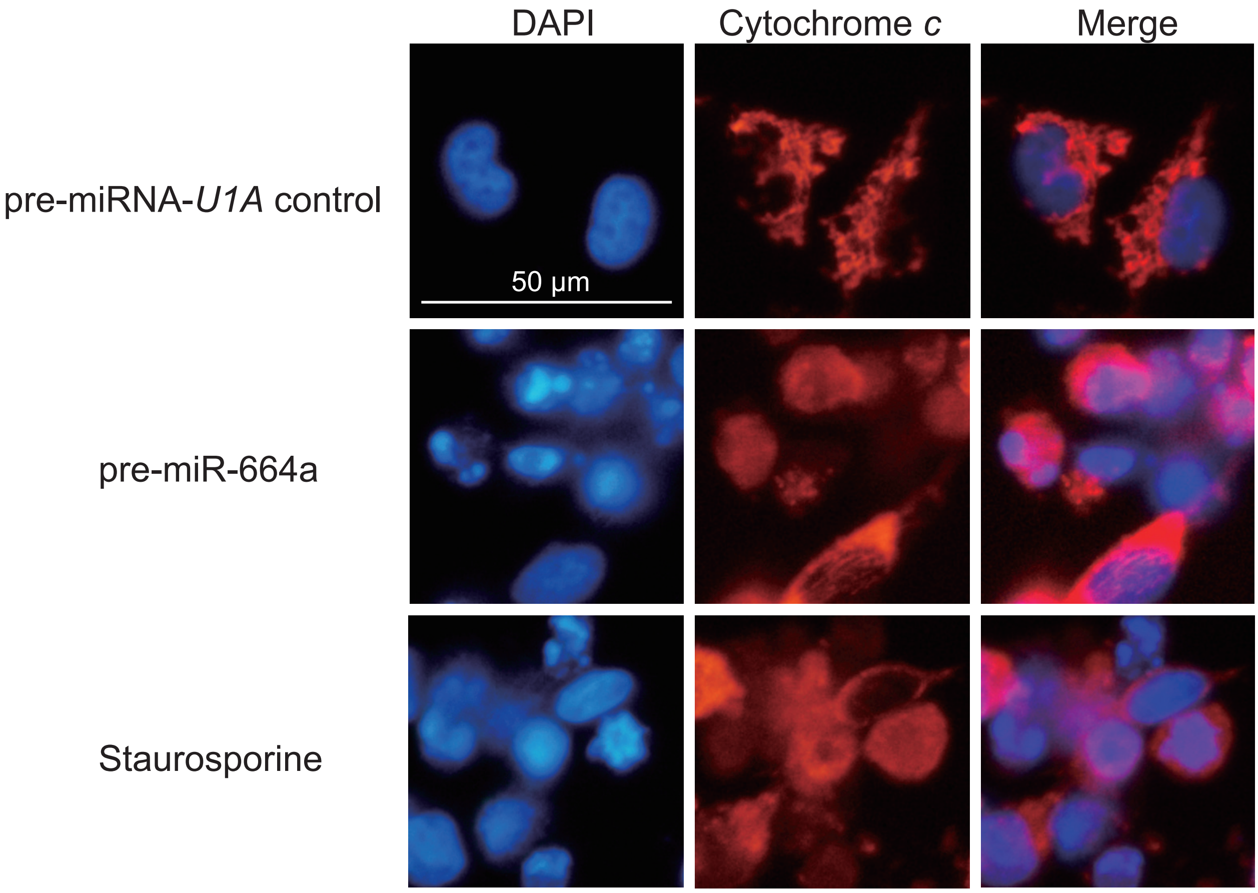

Figure S2. Localization of cytochrome c in the cells treated with pre-miRNA-*U1A* control or pre-miR-664a.

The localization of cytochrome *c* in HeLa cells treated with pre-miRNA-*U1A* control or pre-miR-664a was monitored using immunostaining. Nuclear DNA was stained with DAPI. 0.25 μM staurosporine was used as a positive control.

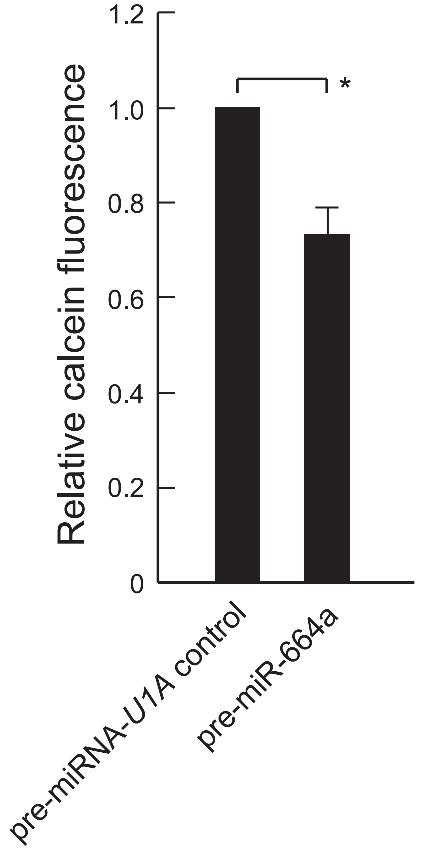

Figure S3. pre-miR-664a induced MPTP opening.

Transfected HeLa cells treated with 1 μM calcein-AM and 5 mM $CoCl_2$ to measure the calcein fluorescence in the mitochondria. Quantification of calcein fluorescence in cells treated with pre-miRNA-*U1A* control or pre-miR-664a. More than 500 cells were counted in each experiment. Data represent the means ± SEM of five independent experiments. *P<0.01; P-values were calculated using one-way ANOVA and Dunnet' s multiple comparisons test.

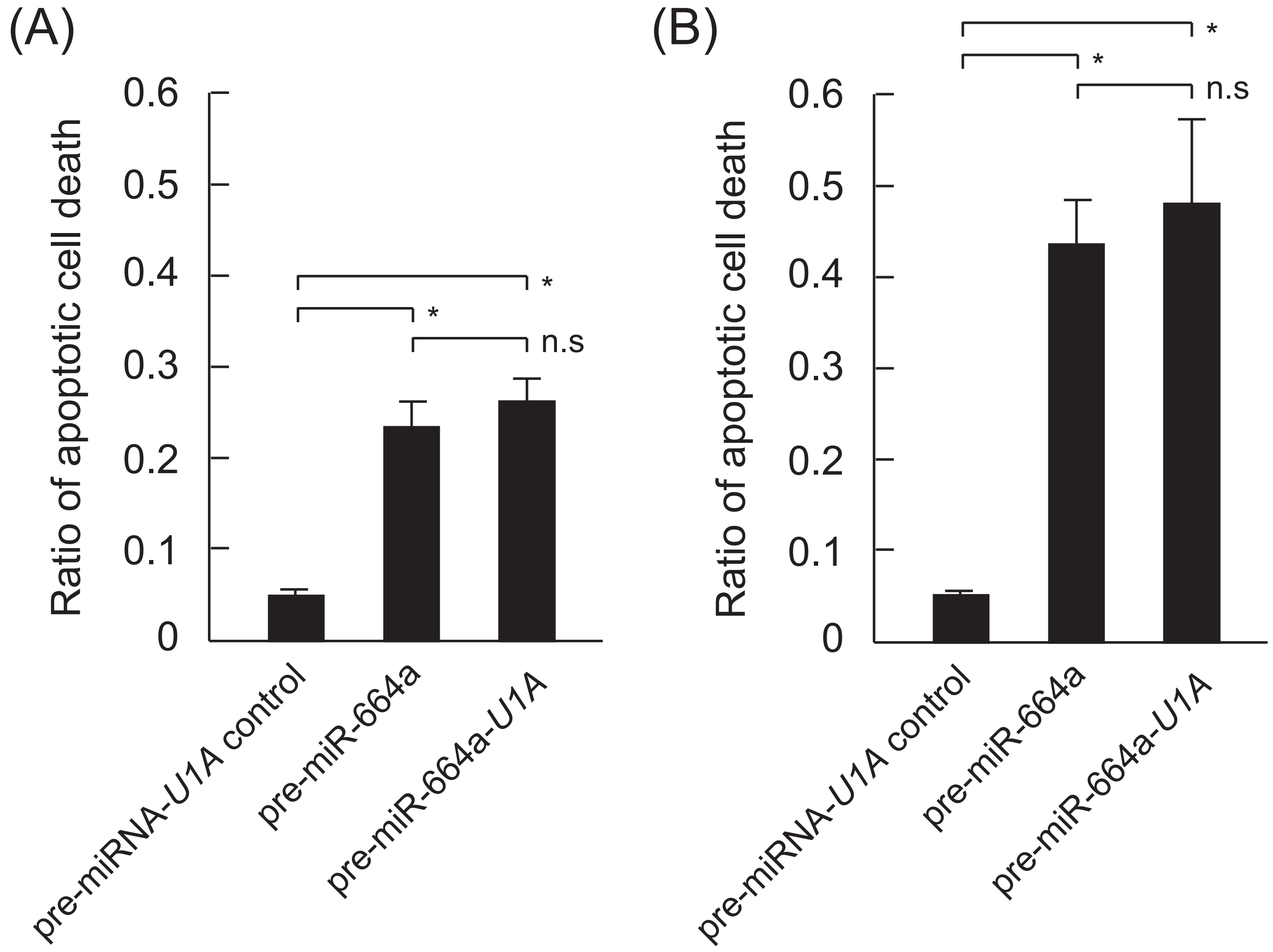

Figure S4. Apoptosis efficiencies by pre-miR-664a and pre-miR-664a-*U1A*.

(A) Apoptosis efficiencies of SH-SY5Y cells transfected with pre-miRNA-*U1A* control, pre-miR-664a and pre-miR-664a-*U1A*. (B) Apoptosis efficiencies of SH-SY5Y cells transfected with pre-miRNA-*U1A* control, pre-miR-664a and pre-miR-664a-*U1A*. Data represent the means ± SEM of four independent experiments. *P<0.01; P-values were calculated using one-way ANOVA and Tukey’ s multiple comparisons test.

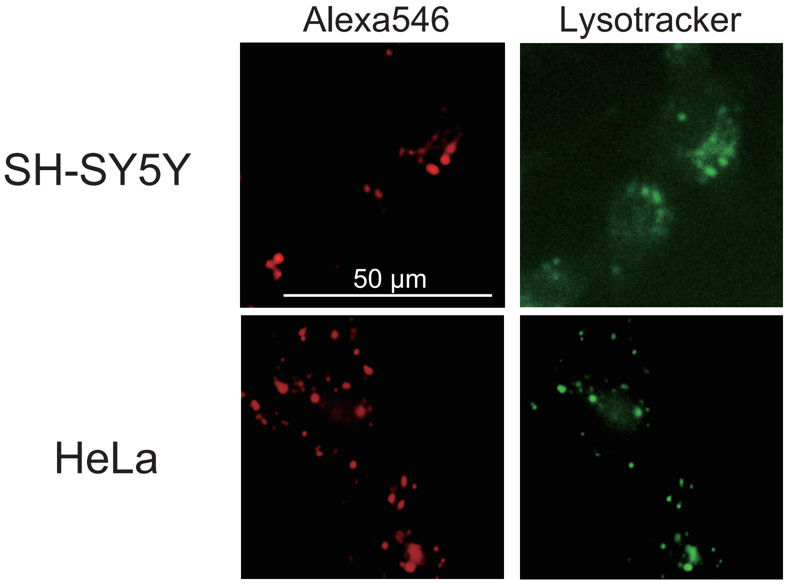

Figure S5. Cellular uptake of TatU1A-Alexa/RNA complexes into endosome by detecting Lysotracker green.

Endosome was detected by 5 μM Lysotracker green in SH-SY5Y cells and by 2 μM Lysotracker green in HeLa cells.

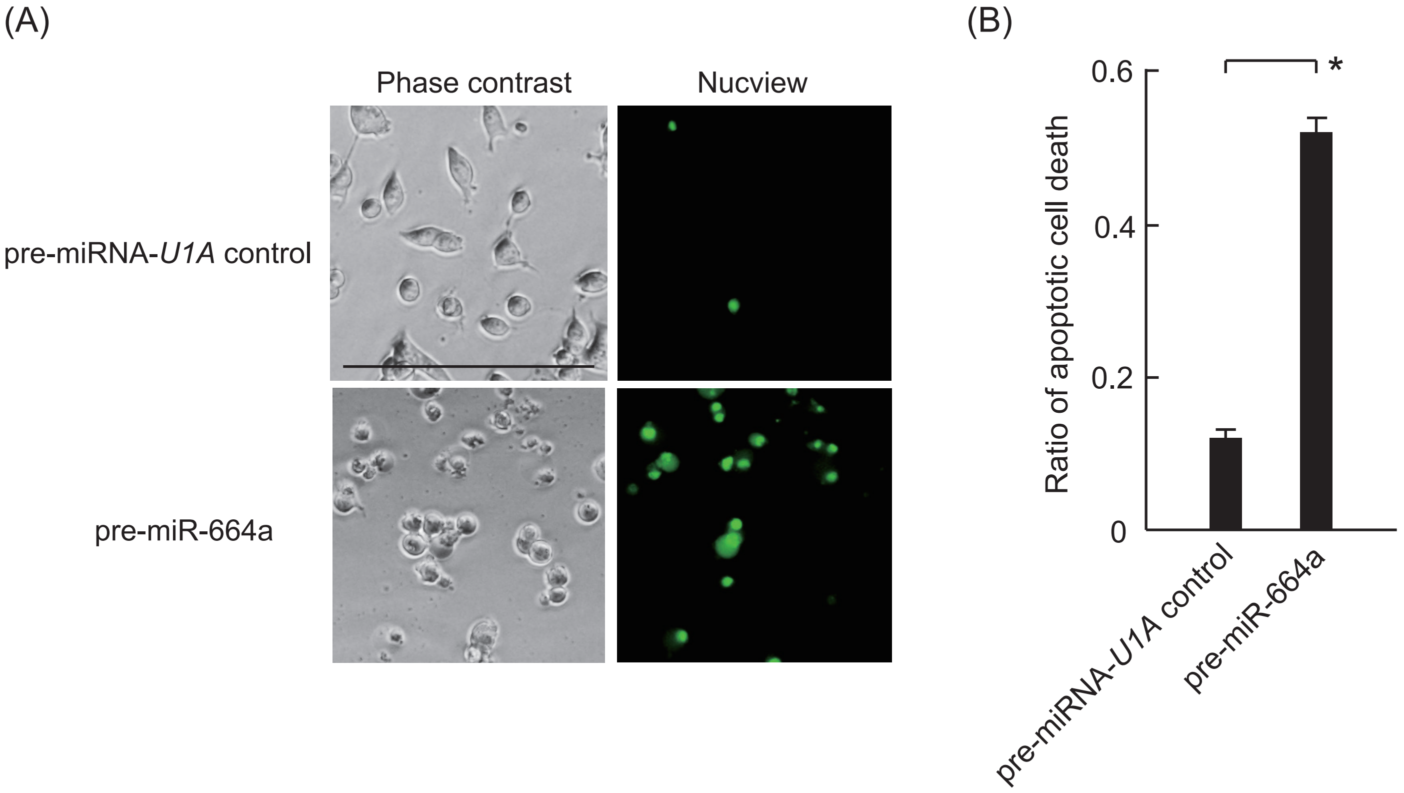

Figure S6. pre-miR-664a delivered by lipofection induces apoptosis in Flp-In-293 cells.

(A) Flp-In-293 cells were transfected with pre-miRNA-*U1A* control or pre-miR-664a using Lipofectamine 3000. Apoptotic cells were detected using NucView 488 caspase assay kit. Scale bars indicate 100 μm. (B) Apoptosis efficiency of Flp-In-293 cells are shown. Data represent the means ± SEM of four independent experiments. *P<0.001; one-way ANOVA and Dunnet' s comparisons test.
